# The new locally endemic genus *Yazdana* (Caryophyllaceae) and patterns of endemism highlight the high conservation priority of the poorly studied Shirkuh Mountains (central Iran)

**DOI:** 10.1111/jse.12575

**Published:** 2020-03-24

**Authors:** Jalil Noroozi, Atefeh Pirani, Hamid Moazzeni, Mohammad Mahmoodi, Golshan Zare, Alireza Noormohammadi, Michael H.J. Barfuss, Michael Suen, Gerald M. Schneeweiss

**Affiliations:** ^1^ Department of Botany and Biodiversity Research University of Vienna Vienna 1030 Austria; ^2^ Department of Biology, Faculty of Sciences Ferdowsi University of Mashhad Mashhad 91779‐48974 Iran; ^3^ Department of Botany, Research Center for Plant Sciences Ferdowsi University of Mashhad Mashhad 91779‐48974 Iran; ^4^ Botany Research Division, Research Institute of Forests and Rangelands Agricultural Research, Education and Extension Organization (AREEO) Tehran 13185‐116 Iran; ^5^ Department of Pharmaceutical Botany, Faculty of Pharmacy Hacettepe University Ankara 06100 Turkey; ^6^ Cologne Excellence Cluster for Cellular Stress Responses in Aging‐Associated Diseases (CECAD) University of Cologne Cologne 50931 Germany

**Keywords:** biogeography, endemism, genus novum, phylogeny, species nova, taxonomy

## Abstract

Although mountain ranges are often recognized as global biodiversity hotspots with a high level of endemism, diversity and biogeographic connections of isolated and weakly explored mountains remain poorly understood. This is also the case for Shirkuh Mts. in central Iran. Here, *Yazdana shirkuhensis* gen. & spec. nov. (Caryophylleae, Caryophyllaceae) is described and illustrated from the high alpine zone of this mountain. Molecular phylogenetic analyses of nuclear and plastid DNA sequence data show that *Y. shirkuhensis* is related to *Cyathophylla* and *Heterochroa* (tribe Caryophylleae). The newly described genus and species accentuate Shirkuh Mts. as a center of endemism, which harbors a high number of narrowly distributed species, mostly in high elevations reaching alpine habitats. As this area is currently not protected, a conservation priority is highlighted for high elevations of Shirkuh Mts.

## 1 Introduction

**Figure 1:**
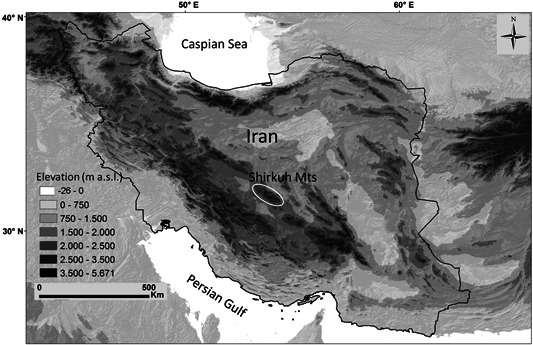
Topographic map of the Iranian Plateau showing the location of the Shirkuh Mts.

Mountains are biodiversity hotspots (Spehn et al., 2011), which harbor a considerable number of endemic species (Barthlott et al., 1996; Körner, 2003), mostly in the alpine zone (Nagy & Grabherr, 2009; Hobohm et al., 2014). Iran is a mountainous country (Fig. 1), and a high proportion of the Iranian flora (74%) is concentrated or even restricted to mountain ranges (Noroozi et al., 2019b). With increasing elevation, the rate of endemism increases, and in spite of the small area size of the alpine zone relative to lower elevations, a considerable number of Iranian endemic species are restricted to this habitat (Noroozi et al., 2018, 2019a, 2019b). Although larger areas of alpine zone can be found in Alborz and Zagros, there are numerous smaller and isolated high mountains in different parts of the country. One of these isolated mountain systems is Shirkuh in central Iran (4050 m a.s.l. at the highest peak; Fig. 1), west of the city of Yazd. Together with the Kerman massif, Shirkuh Mts. have recently been identified as an area of endemism (Noroozi et al., 2019b). However, the high elevations of the Yazd‐Kerman Massifs have been poorly investigated, and in remote regions, it is still possible to find taxa new to the regional flora or even new to science (e.g., Ajani et al., 2010; Noroozi et al., 2010; Rajaei et al., 2011; Mahmoodi et al., 2013; Moazzeni et al., 2014, 2016; Doostmohammadi & Kilian, 2017). Indeed, during a field trip to the highest summit of Shirkuh Mts. in summer 2012, an annual species of Caryophyllaceae was collected close to the summit. It could neither be determined with available floras nor could it be unambiguously assigned to any of the Iranian genera of the family, suggesting that it belongs to a new taxon.

Caryophyllaceae is a large mainly Holarctic family of approximately 3000 species of herbs and subshrubs, with its diversity center in the Mediterranean and the adjacent Irano‐Turanian region (Bittrich, 1993; Ghaffari, 2004; Hernández‐Ledesma et al., 2015). Recent molecular investigations of the family have shown that many of the traditionally defined genera are not monophyletic (Dillenberger & Kadereit, 2014; Pirani et al., 2014; Sadeghian et al., 2015; Madhani et al., 2018). This is also the case for the tribe Caryophylleae, where, based on morphology (connate sepals, stipitate ovary, and presence of two styles), the plant from the Shirkuh Mts. was suspected to belong to. Madhani et al. (2018), revising tribe Caryophylleae, described three new genera and resurrected one genus. Thus, currently the tribe contains 14 genera (*Acanthophyllum* C.A.Mey., *Balkana* Madhani & Zarre, *Bolanthus* (Ser.) Rchb., *Cyathophylla* Bocquet & Strid, *Dianthus* L., *Diaphanoptera* Rech.f., *Graecobolanthus* Madhani & Rabeler, *Gypsophila* L., *Heterochroa* Bunge, *Petroana* Madhani & Zarre, *Petrorhagia* (Ser.) Link, *Psammophiliella* Ikonn., *Psammosilene* W. C. Wu & C. Y. Wu, *Saponaria* L.), of which seven are found in Iran (*Acanthophyllum, Cyathophylla*, *Dianthus, Diaphanoptera, Gypsophila, Petrorhagia, Saponaria*; Madhani et al., 2018).

As morphological data did not permit assignment of the plants from Shirkuh Mts. to any of the currently recognized genera, we used molecular phylogenetic data to place this taxon within the phylogenetic framework of the tribe Caryophylleae established by Pirani et al. (2014) and Madhani et al. (2018). Hence, by determining the phylogenetic position of the new taxon based on molecular data, we wanted to clarify its taxonomic position. In light of the obtained taxonomic results (i.e., description of a new genus and species: see Section 3) and a lack of a phytogeographic study of endemics in the Shirkuh Mts., we subsequently addressed the floristic relationships of these mountains to other mountain ranges of the Iranian Plateau on the basis of distribution patterns of Iranian endemics.

## 2 Material and Methods

### 2.1 Study area

**Figure 2:**
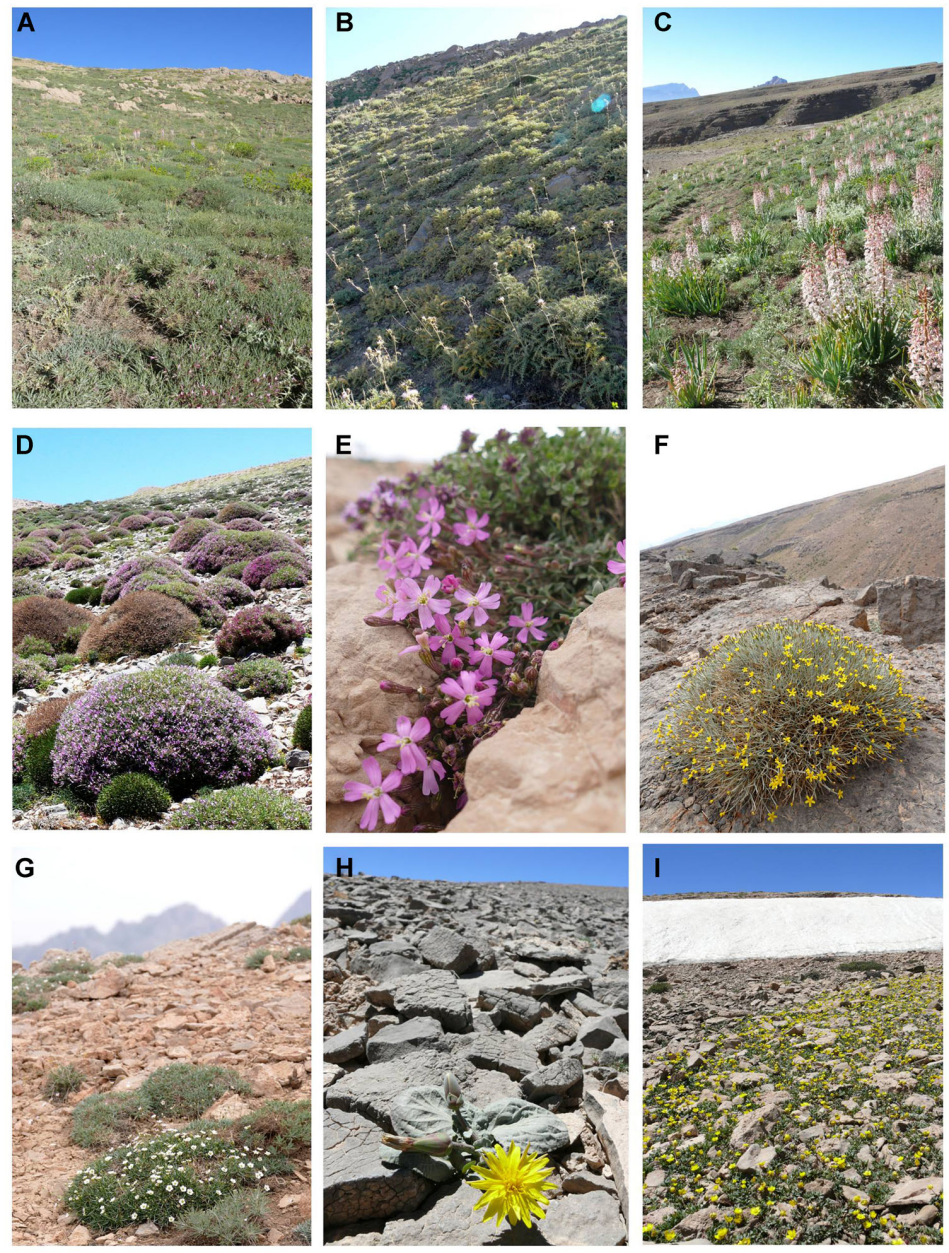
Some of the characteristic species of the dominant vegetation types in the alpine zone (above 3500 m a.s.l.) of Shirkuh Mts. **A,** *Astragalus microphysa* Boiss., Fabaceae (3600–4050 m a.s.l.). **B,** *Cousinia lasiolepis* Boiss., Asteraceae (3600–4050 m a.s.l.). **C,** *Eremurus persicus* Boiss., Asphodelaceae (3600–3900 m a.s.l.). **D,** *Onobrychis cornuta* (L.) Desv., Fabaceae (3600–4050 m a.s.l.). **E,** *Silene nurensis* Boiss. & Hausskn., Caryophyllaceae (3600–4050 m a.s.l.). **F,** *Scorzonera intricata* Boiss., Asteraceae (3500–4050 m a.s.l.). **G,** *Arenaria persica* Boiss., Caryophyllaceae (3700–4050 m a.s.l.). **H,** *Scorzonera paradoxa* Fisch. & C.A.Mey. (3600–4050 m a.s.l.). **I,** *Ranunculus eriorrhizus* Boiss. & Buhse, Ranunculaceae (3700–4050 m a.s.l.). Photos by J. Noroozi.

Shirkuh Mts. (31.380° to 31.880° N; 53.700° to 54.430° E) are located in the southern part of the Irano‐Turanian region, with a dry and continental climate (Zohary, 1973; Ebrahimi et al., 2010; Djamali et al., 2012), and annual rainfall of 350–400 mm mainly from October to May (Grunert et al., 1978). However, during the Pleistocene ice ages, these mountains were locally glaciated (Haars et al., 1974). Based on the Global Bioclimatic Classification System developed by Rivas‐Martínez et al. (1997, 1999), Shirkuh Mts. belong to the Mediterranean Xeric continental, which is surrounded by Mediterranean Desertic continental (Djamali et al., 2011). Shirkuh Mts. are a part of the Yazd‐Kerman area of endemism within the Irano‐Anatolian biodiversity hotspot (Noroozi et al., 2019b), and they have recently been identified as a priority conservation gap (i.e., a center of endemism that is not or only marginally covered by protected areas; Noroozi et al., 2019a). Steppe vegetation dominates across all elevation zones. The alpine zone is above ca. 3500 m a.s.l. and, similar to other alpine habitats of the region (Noroozi et al., 2008), is covered by thorn‐cushion grasslands (Figs. 2A–2D), rock habitats (Figs. 2E, 2F), screes (Figs. 2G, 2H), and snowbeds (Fig. 2I).

### 2.2 Plant material

Plant material of the new taxon was collected in early July 2012 and, in the course of a trip dedicated to re‐collect this species, in mid July 2019. For the molecular investigation, leaves of six individuals (one collected in 2012, taken as a herbarium voucher, and five collected in 2019, stored in silica‐gel) were used. For detailed morphological investigation, 18 individuals (3 from 2012 and 15 from 2019) were collected as vouchers.

### 2.3 Molecular phylogenetic analysis

Total genomic DNA was extracted using the DNeasy Plant Mini Kit (QIAGEN, Hilden, Germany) according to the manufacturer's protocol. The plastid *rps16* intron and the nuclear ribosomal ITS (internal transcribed spacer) regions were amplified and sequenced using the primers *rps*F and *rps*R2 (Oxelman et al., 1997), and ITS18Sfa (ITS18Scsf, 5′‐GAA TGG TCC GGT GAA GTG TTC G‐3′) and ITS26Sra (ITS26Scsr, 5′‐GGA CGC TTC TCC AGA CTA CAA TTC G‐3′; Barfuss, 2012), respectively. For ITS, two additional sequencing reactions were performed using the internal primers ITS5.8Sfa (ITS5.8Scf, 5′‐GAC TCT CGG CAA CGG ATA TCT CG‐3′) and ITS5.8Sra (ITS5.8Scsr, 5′‐GAT GCG TGA CGC CCA GGC AG‐3′), which are located in the 5.8S region (Barfuss, 2012). Wet lab procedures principally follow the protocol by Ehrendorfer et al. (2018), but amplification was done with 2× Phusion Green Hot Start II High‐Fidelity PCR Master Mix (Thermo Scientific, Vienna, Austria) using a standard protocol with optimized annealing temperatures (*rps16*: 68 °C; ITS: 70 °C) and the addition of 3% DMSO (Sigma‐Aldrich) for ITS.

Sequences were trimmed and assembled in Geneious 6.1.2 (https://www.geneious.com). The 12 newly obtained sequences (six for each marker) were added to the Caryophylleae data set of Madhani et al. (2018) and aligned using the MUSCLE plug‐in in Geneious 6.1.2. Thus, the ITS data set contained 132 accessions representing 113 species and the *rps16* data set contained 119 accessions representing 86 species (Doc. S1). Following Madhani et al. (2018), the genus *Silene* (five species sampled) was selected as outgroup. Nuclear and plastid DNA sequence data were analyzed separately and jointly. Combinability of the markers was assessed with the incongruence length difference (ILD) test (Farris et al., 1995) implemented as the partition homogeneity test in PAUP* 4.a164 (Swofford, 2002) at the CIPRES portal using a full heuristic search, 10 random taxon addition replicates, tree bisection and reconnection (TBR) branch swapping, and with MaxTrees set to 100. Moreover, visual inspection of nodes in the separate analyses did not show any mutually strongly contradicted nodes (i.e, with bootstrap support values of at least 70 and posterior probabilities of at least 0.95).

The three datasets (ITS, *rps16*, and combined datasets) were analyzed using maximum likelihood (ML) and Bayesian methods. The best‐fit substitution models for the ITS and the *rps16* data, determined using the Akaike Information Criterion (AIC) as implemented in jModelTest 2.1.4 (Darriba et al., 2012), were the GTR + I + G and the GTR + G model, respectively. Maximum likelihood analysis was carried out on the RAxML web server (RAxML‐HPC2 on XSEDE; available at the CIPRES portal: http://www.phylo.org/index.php/portal/) using 1000 bootstrap replicates, obtained by the rapid bootstrap algorithm (Stamatakis et al., 2008). Bayesian inference (BI) analyses were performed using MrBayes 3.1.2 (Huelsenbeck & Ronquist, 2001) using default prior settings and a random starting tree. The analysis consisted of four parallel runs, each with three heated chains and one cold chain that were run for 10 million generations, with each sampling every 1000 generations. The quality of the analysis was checked by comparing likelihood values and parameter estimates from different runs in Tracer 1.6 (Rambaut et al., 2014) and by average standard deviations of split frequencies (less than 0.01), and the first 25% of the trees were discarded as burn‐in. The remaining trees were summarized in a 50% majority‐rule consensus tree.

### 2.4 Morphological analysis

Morphological characteristics such as plant habit and the color of the flowers were investigated in the natural habitat; measurements and the study of micromorphological characteristics were conducted on the 18 collected individuals (see Section 2.2). Morphological data for *Cyathophylla* and *Heterochroa*, the closest relatives of the new taxon, were extracted from literature (Schischkin, 1936; Barkoudah, 1962; Davis, 1966; Strid, 1986; Rechinger & Schiman‐Czeika, 1988; Madhani et al., 2018).

### 2.5 Biogeography of Shirkuh Mts.

All endemic species of the Iranian Plateau present (also) in Shirkuh Mts. at elevations above 1400 m a.s.l. were recorded, and their distribution patterns in different mountain ranges of Iran (which are well associated with the identified areas of endemism of this region according to Noroozi et al., 2018, 2019b) were analyzed. These data were extracted from the database of endemic vascular plant species of Iran (Noroozi et al., 2019b). The distribution patterns were illustrated by ArcGIS 10 (Esri, Redlands, CA, USA; Jenness, 2013).

## 3 Results and Discussion

### 3.1 Phylogenetic analyses and taxonomic treatment

**Figure 3:**
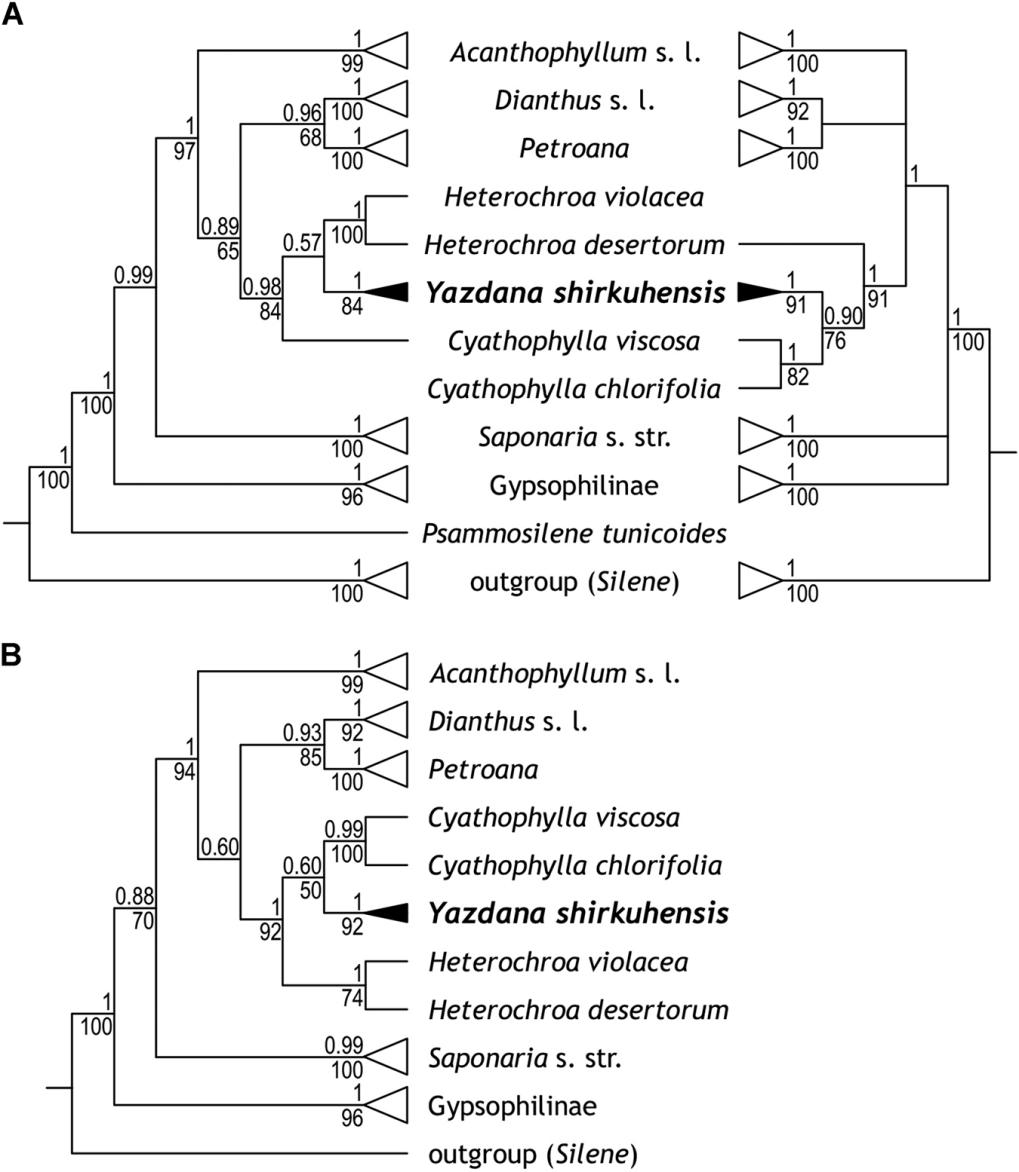
Majority‐rule consensus trees inferred from Bayesian analysis of **A,** ITS (left) and *rps16* data (right) and **B,** of the combined data from tribe Caryophylleae; both the clade of six accessions of *Yazdana shirkuhensis* (black triangles) and the clades not pertinent for the position of *Yazdana* (white triangles) are collapsed (see Madhani et al., 2018, for details on these latter lineages). Numbers above branches indicate posterior probabilities and those below indicate maximum likelihood bootstrap values.

The newly obtained sequences are available in GenBank under accession numbers MK637517 and MN381230–MN381234 for ITS and MK651077 and MN417289–MN417292 for *rps16*. The newly generated ITS sequences did not contain any polymorphic sites. All accessions of the new taxon form a clade (bootstrap support [BS]/posterior probability [PP] of 84/1.00 from ITS; BS/PP of 91/1.00 from *rps16*; BS/PP of 92/1.00 from the combined dataset; Doc. S2). The new taxon is placed in tribe Caryophylleae with strong support as closely related to *Cyathophylla* and *Heterochroa* (BS/PP of 84/0.98 from ITS; BS/PP of 91/1.00 from *rps16*; BS/PP of 92/1.00 from the combined dataset; Fig. 3). Whether the new taxon is sister to *Heterochroa*, as inferred by ITS, or to *Cyathophylla*, as inferred by *rps16* and the combined data, remained unclear due to low support values (Fig. 3). Both genera belong to subtribe Caryophyllineae, whose internal relationships were barely known until the recent molecular study of Madhani et al. (2018). This is also the case for *Heterochroa* and *Cyathophylla*, which were previously classified under *Gypsophila* and *Saponaria*, respectively (see Madhani et al., 2018, for details on their taxonomic history). *Heterochroa* includes six perennial species distributed in Kazakhstan, Mongolia, N China, and Russia, whereas *Cyathophylla* comprises two annual species distributed from Greece to Turkmenistan (Madhani et al., 2018). However, several of these species are only poorly known. The new species from Shirkuh Mts. has dark purple stems and calyces covered with long‐stemmed glandular hairs, only slightly clawed bicolored and emarginate petals. As it differs morphologically from both *Heterochroa* and *Cyathophylla* in habit, coloration, leaf shape and petal shape, and color, and as the phylogenetic position of the new taxon relative to these two genera remains unclear (contradicting, yet insufficiently supported relationships inferred from the two markers: Fig. 3), the plant from Shirkuh Mts. is described here as a new species and placed in a new genus.

**Figure 4:**
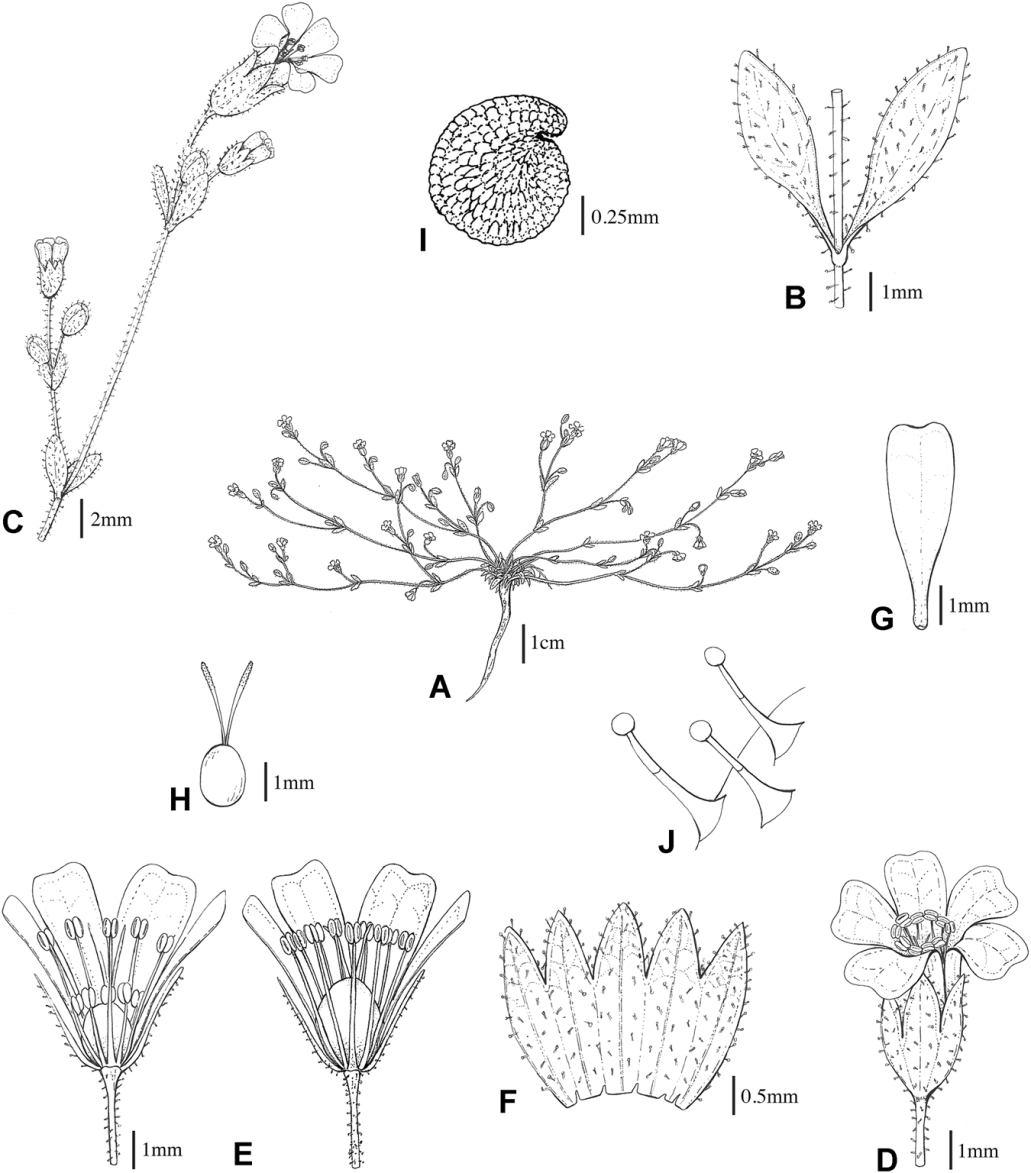
*Yazdana shirkuhensis*. **A,** Habit. **B,** Leaf attachment. **C,** Inflorescence. **D,** Flower. **E,** Longitudinal sections of early (left) and late (right) flower. **F,** Calyx. **G,** Petal. **H,** Pistil. **I,** Seed. **J,** multicellular glandular hairs. Drawings by G. Zare.

**Figure 5:**
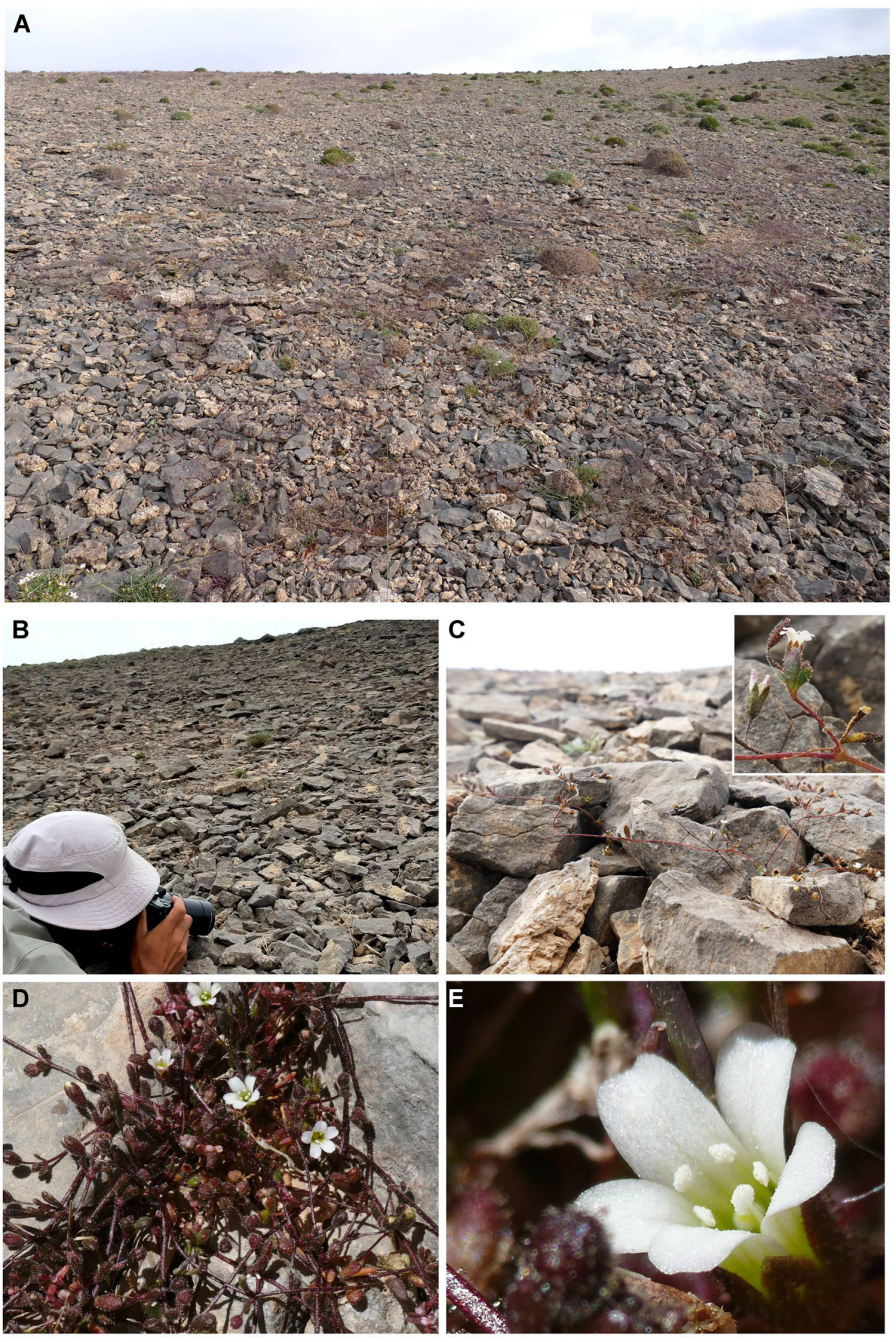
**A, B,** Habitat and **C, D, E,** habit of *Yazdana shirkuhensis*. The plants are found only on the northern slope of the highest summit of Shirkuh Mts. (3950–4050 m a.s.l.), where they grow on limestone screes. Photos by J. Noroozi.

***Yazdana*** A.Pirani & Noroozi, **gen. nov.** (Figs. 4, 5)

Type: *Yazdana shirkuhensis* A.Pirani & Noroozi, sp. nov. Iran, Yazd, Baft, Dehbala village, near the highest summit of Shirkuh Mts., 31.610° N, 54.068° E, 3950–4050 m a.s.l., on limestone screes, 14 July 2019, *J. Noroozi 4003* (holotype, TARI; isotypes, FUMH, IRAN, HUM, W, WU). Additional specimens: Iran, Yazd, Shirkuh Mt., 31.610° N, 54.068° E, 4000 m a.s.l., on scree grounds, 5 July 2012, *J. Noroozi 2827* (WU).

Diagnosis: This monotypic genus is similar to *Cyathophylla* and *Heterochroa*, but it differs from *Cyathophylla* by non‐perfoliate leaves, bicolored petals, and capsules ±enclosed in the calyx, and from *Heterochroa* in being annual, possessing dark purple stems, having basal spathulate leaves and capsules ±enclosed in the calyx. A comparison among the three genera is provided in Table 1.

**Table 1:** Comparison of morphological characteristics of *Yazdana* and allied genera (Rechinger & Schiman‐Czeika, 1988; Madhani et al., 2018)

	*Cyathophylla*	*Heterochroa*	*Yazdana*
Habit	Annual	Perennial cespitose	Annual
Indumentum	Glabrous or covered with long glandular multicellular and sessile glandular hairs	Glabrous or covered with long multicellular glandular hairs	Covered with long multicellular glandular hairs
Basal leaves	In a rosette, ovate or broadly spathulate, obtuse	Lacking	In a rosette, spathulate
Cauline leaves	Linear‐lanceolate, ovate to rounded	Linear, linear‐subulate, linear‐lanceolate, lanceolate, or ovate	Oblong‐lanceolate
Cauline leaf size	4–10 × 1–5 mm	3–10 × 0.5–4 mm	3–5.5 × 1.5–2 mm
Calyx	5 or 15‐nerved	5‐nerved	10‐nerved
Petal color	Concolorous (rose or pink)	Concolorous (white to purple)	Bicolorous (greenish at base, white to white tinged with purple at upper part)
Capsule length	Exceeding the calyx	Equaling or exceeding the calyx	shorter or subequal to the calyx
Seed shape	Roundish	Reniform‐oblong	Reniform to reniform‐roundish

Note: The generic and the specific names are published here simultaneously via a single diagnosis (descriptio generico‐specifica; see Art. 38.5 of the Shenzhen Code: Turland et al., 2018).

Description: Annual herb, up to 17 cm high, densely branched from the base, covered with ±dense long glandular hairs, glabrescent with age. Stems prostrate, dark purple. Leaves opposite, basal leaves in rosette, spathulate, ca. 5–10 × 1–2 mm, cauline leaves oblong‐lanceolate, ca. 3–5.5 × 1.5–2 mm. Flowers in lax monochasial inflorescences; pedicellate, pedicels 3–15 mm long; sepals five, 3–3.5 mm long, connate at lower 2/3; calyx 10‐nerved, cylindrical, dark purple to dark green, covered with glandular hairs; petals 5, bicolored (white to white tinged with purple at the upper 2/3 and greenish at the lower third), emarginate, 4–4.5 mm long, only slightly clawed; stamens 10, enclosed, 3.5–4 mm, developing non‐simultaneously (episepalous stamens reach full length distinctly before the epipetalous stamens); styles 2, 1.5–2 mm; ovary with a short gynophore; ovules 6–10; capsules oblong, slightly shorter than or subequal to the calyx, opening with 4 valves; seeds 1–8, reniform to reniform‐roundish, black, 1 × 0.8 mm.

Etymology: The generic name refers to the city of Yazd in central Iran, whereas the specific name refers to Shirkuh Mts. in the vicinity of Yazd.

Distribution: It is found only in Shirkuh Mts., immediately below the highest summit (4050 m a.s.l.) on the northern slope.

Habitat: This species grows on limestone screes in the high alpine zone. The accompanying species of *Yazdana*, based on six vegetation plots (each of 5 × 5 m), are presented in Table 2. Although the number of annual species is usually very low in the high alpine zone of Iranian mountains (Noroozi et al., 2008), there are several annual and geographically restricted species in these small scree patches. A similar situation can be found near the summit of Hezar Mt. in Kerman, where recently a new annual *Senecio* has been discovered (Noroozi et al., 2010).

**Table 2:** Accompanying species of *Yazdana shirkuhensis* based on six vegetation plots (5 × 5 m) sorted by life form

Species	Family	Life form	Distribution
*Acantholimon modestum* Bornm. ex Rech.f. & Schiman‐Czeika	Plumbaginaceae	Thorn‐cushion	Yazd‐Kerman
*Acanthophyllum glandulosum* Buhse ex Boiss.	Caryophyllaceae	Thorn‐cushion	Iran, Hindu Kush, C Asia
*Arenaria persica* Boiss.	Caryophyllaceae	Thorn‐cushion	Zagros and Shirkuh Mts.
*Alyssum muelleri* Boiss. & Buhse	Brassicaceae	Hemicryptophyte	Iran
*Asperula glomerata* (M.Bieb.) Griseb. subsp. *filiformis* (Bornm.) Ehrend. & Schönb.‐Tem	Rubiaceae	Hemicryptophyte	Zagros and Yazd‐Kerman
*Crepis heterotricha* DC.	Asteraceae	Hemicryptophyte	Iran
*Cousinia lasiolepis* Boiss.	Asteraceae	Hemicryptophyte	Zagros and Shirkuh Mts.
*Elymus longearistatus* (Boiss.) Tzvelev	Poaceae	Hemicryptophyte	SW Asia
*Oxytropis shirkuhi* Vassilcz.	Fabaceae	Hemicryptophyte	Shirkuh Mts.
*Piptatherum laterale* (Regel) Roshev.	Poaceae	Hemicryptophyte	SW and C Asia
*Pseudocamelina camelinae* N. Busch	Brassicaceae	Hemicryptophyte	Zagros and Yazd‐Kerman
*Scorzonera paradoxa* Fisch. & C.A.Mey.	Asteraceae	Hemicryptophyte	SW Asia
*Silene nurensis* Boiss. & Hausskn.	Caryophyllaceae	Hemicryptophyte	Zagros and Yazd‐Kerman
*Stachys obtusicrena* Boiss.	Lamiaceae	Hemicryptophyte	Zagros and Shirkuh Mts.
*Trachydium depressum* (Boiss.) Boiss.	Apiaceae	Hemicryptophyte	SW Asia
*Zerdana anchonioides* Boiss. (monotypic genus)	Brassicaceae	Hemicryptophyte	Zagros and Yazd‐Kerman
*Allium kotschyi* Boiss.	Alliaceae	Geophyte	Zagros and Shirkuh Mts.
*Bromus gracillimus* Bunge	Poaceae	Annual	SW Asia
*Chaenorhinum grossecostatum* Speta	Plantaginaceae	Annual	Yazd‐Kerman
*Polygonum molliaeforme* Boiss.	Polygonaceae	Annual	SW Asia
*Sedum kotschyanum* Boiss.	Crassulaceae	Annual	Zagros and Yazd‐Kerman
*Senecio kotschyanus* Boiss.	Asteraceae	Annual	Yazd‐Kerman

Conservation status: The new species is known only from the type locality. In the year 2012, only a few specimens were collected from a small plot (5 × 5 m) without particular attention to the population size. In 2019, the location was well explored to find more individuals of the species and to make more detailed observations on its ecology and accompanying species. The species grows only in the northern slope and in a few scree patches from 3950 up to 4050 m a.s.l. The size of the population was estimated to have been between 100 to 200 individuals in this year. Its conservation status is, thus, given as Critically Endangered (CE, i.e., facing an extremely high risk of extinction in the wild) according to IUCN criterion B (geographic range; IUCN, 2012). Generally, alpine and subnival species are under high pressure due to ongoing global warming (Dullinger et al., 2012; Pauli et al., 2012). In the absence of higher elevations or alternative habitats for this species to shift up, with ongoing global warming, it is possibly even more strongly threatened.

Identification key: To allow *Yazdana* to be distinguished from other genera of tribe Caryophylleae, we present a generic key modified from the one provided by Madhani et al. (2018; modifications are indicated in bold):

1a. Seeds peltate, with central (facial) hilum; embryo straight………………………………………………………………2
1b. Seeds reniform, reniform‐oblong, **reniform‐roundish** or comma‐shaped, with lateral hilum; embryo curved or hook‐shaped………………………………………………………………….5
2a. Leaves with short petiole, ovate; stamens 5; capsules membranous, nearly indehiscent……………..*Psammosilene*
2b. Leaves sessile, linear, subulate, grass‐like; stamens (5)10; capsules papery, dehiscent…………………………………..3
3a. Calyx without membranous commissures, with 35 or more veins, rarely 5‐15‐nerved (cf. *Velezia*); calyx tube long tubular, teeth straight……………………………………………………………………………………….*Dianthus* (incl. *Velezia*)
3b. Calyx with membranous commissures, with 5‐15 veins; calyx tube variously shaped, if tubular the teeth recurved to deflexed………………………………………………..4
4a. Seeds >1.5 mm, with thin margin, smooth on surface…………………………*Dianthus* (incl. *Petrorhagia* p.p)
4b. Seeds <1.5 mm, with thickened margin, reticulate on surface……………………………………………………..*Petrorhagia*
5a. Seeds comma‐shaped (or oblong), with hook‐shaped embryo……………………………………………………………………6
5b. Seeds reniform, **reniform‐roundish**, or reniform‐oblong, embryo curved…………………………………………….7
6a. Petals turning abruptly downward and becoming clearly deflexed (Greece)……………………*Graecobolanthus*
6b. Petals recurved gradually (Turkey to the coastal mountains of Syria, Lebanon and Palestine)……………………………………………………………*Bolanthus* (incl. *Phrynella*)
7a. Calyx bladdery inflated, or turbinate, constricted at teeth, commissural region membranous hyaline, sometimes wing‐like……………………………………..*Diaphanoptera*
7b. Calyx campanulate, **cylindrical, or** tubular, if inflated, commissural regions papery or leafy and main veins with leafy wings……………………………………………………….8
8a. Bracteoles present, leafy, papery or rarely membranous; calyx papery in texture or only membranous at intervals…………………………………………….*Acanthophyllum*
8b. Bracteoles absent…………………………………………………….9
9a. Calyx bladdery inflated, nerves prominent and thick, costate, or winged, midveins 5; bracteoles membranous hyaline……………………………………..*Gypsophila* (cf. *Vaccaria*)
9b. Calyx tubular, campanulate, **cylindrical**, or obconical, not much inflated, lateral nerves obscure, not prominent and thick, midveins 5 or more; bracteoles absent……………..10
10a. Calyx obscurely nerved or with 15‐25 nerves, commissures absent or present; petals inconspicuous, or clawed, mostly with appendages……………………………..11
10b. Calyx 5‐**10** nerved, with membranous commissures; petals not or only indistinctly clawed, without appendages…………………………………………………………………………12
11a. Plants annual; inflorescences congested; capsule slightly longer than the calyx; coronal scales absent…………………………………………………….*Cyathophylla*
11b. Plants annual, biennial or perennial; inflorescences usually lax; capsule mostly shorter than the calyx; coronal scales mostly present………………………*Saponaria*
12a. Leaves fleshy, spathulate; flowers very small: calyx <4mm, corolla<5 mm; seed testa with swollen cells tuberculate on periclinal wall, testa cells polygonal‐oblong, moderately elongated (Iberian Peninsula, Socotra)……………………………………………………….*Petroana*
12b. Leaves not fleshy or subfleshy, linear, **lanceolate**, or ovate; flowers small or large; seed testa variously shaped, with or without tubercles……………………………13
13a. **Petals bicolored……………………………………………………..14**
13b. **Petals always concolored, variously colored; leaves slender, in few species triquetrous, then the plants mostly caespitose, paired at nodes………………………….15**
14a. **Petals red on the outer surface, white or pink on the inner surface; leaves triquetrous, mostly 3 or 4 at each node (Albania, Serbia, Bosnia)………………………..** ***Balkana***
14b. **Petals greenish at base, white to white tinged with purple at apex, leaves slender, paired at each node (Iran)…………………………………………………………….*Yazdana***
15a. The stigmatic surface terminal; ovules fewer than 24…………………………………………………………………..16
15b. The stigmatic surface extending along the inner side of styles; ovules 24‐36………………………………………………..20
16a. Stem nodes with small lateral shoots in leaf axils giving a verticillate appearance; leaves acerose, spiny, or terminating to a spine………………………..*Acanthophyllum*
16b. Lateral shoots in leaf axils absent; leaves not spiny except in *G. acantholimoides* and *G. pinifolia*……………..17
17a. Capsules shorter than the calyx……………………………….18
17b. Capsules exceeding the calyx…………………………………..19
18a. Plants annual, shorter than 10 cm, covered by long glandular hairs………………………..*Bolanthus confertifolius*
18b. Plants perennial, if annual then taller than 10 cm and glandular hairs absent or short……………………*Gypsophila*
19a. Plants perennial; capsules ±indehiscent…………………………………………………*Acanthophyllum* (cf. *A. cerastioides*)
19b. Plants annual or perennial; capsules dehiscent…………20
20a. Calyx without membranous commissural intervals or with very narrow ones, calcium oxalate crystals absent; stamens shorter than the petals……………….*Heterochroa*
20b. Calyx with membranous commissural intervals encompassing calcium oxalate crystals; stamens longer (or sometimes shorter) than petals………………………………21
21a. Annual plants with fibrous roots, puberulent below and glabrous in inflorescence (subcosmopolitan, absent in Australia and New Zealand)………………. *Psammophiliella*
21b. Annual or perennial plants with tap root, variously hairy………………………………………………………….*Gypsophila*

### 3.2 Plant biogeography of Shirkuh Mts.

**Figure 6:**
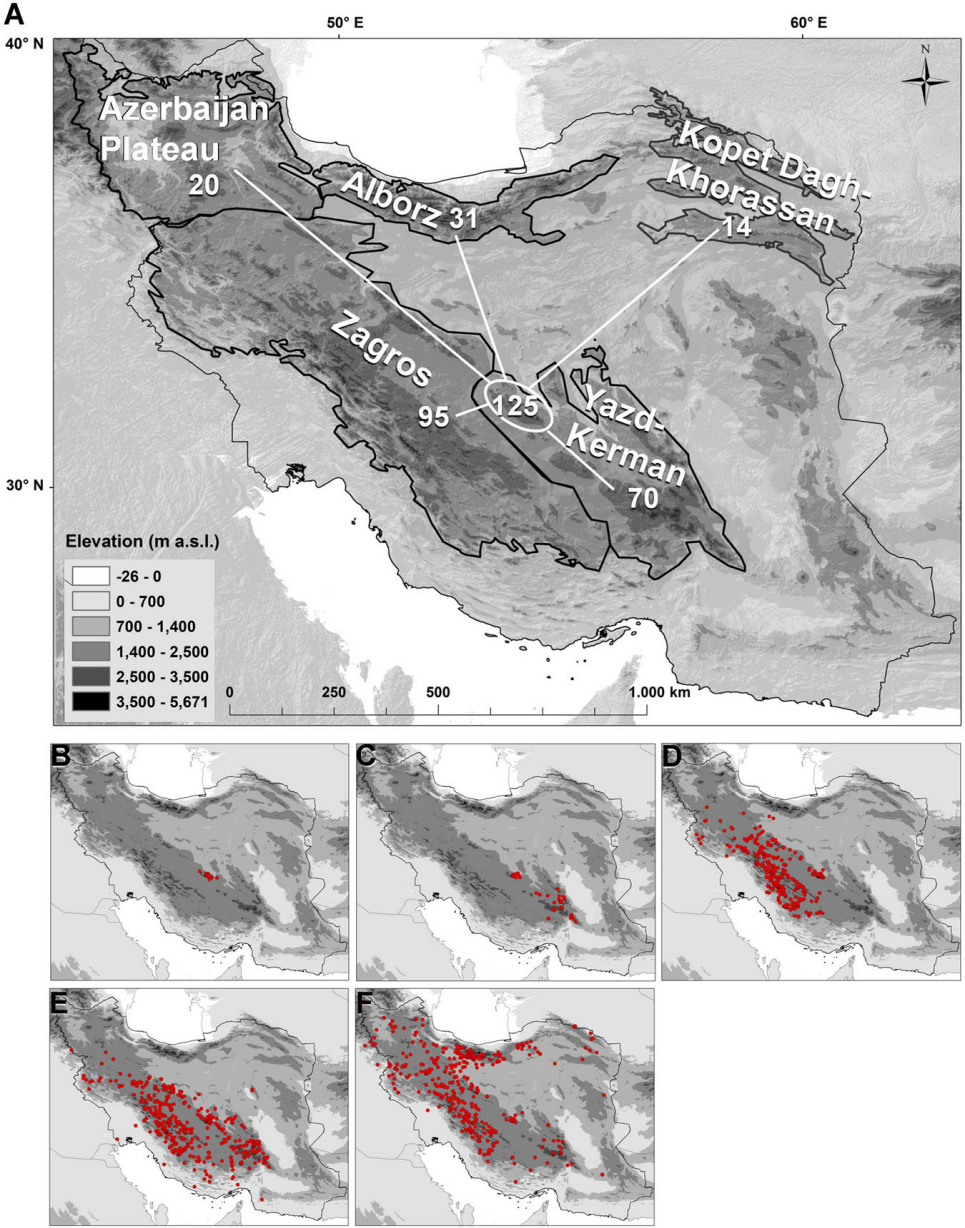
**A,** Floristic relationships, based on the endemic flora of Iran, of Shirkuh Mts. to the areas of endemism of the Iranian Plateau (the numbers written in each area are species shared with Shirkuh Mts.) and (**B–F**) characteristic distribution patterns. **B,** locally endemic to Shirkuh Mts. **C,** endemic to Yazd‐Kerman area. **D,** endemic to the Shirkuh Mts. and to Zagros. **E,** endemic to the entire Yazd‐Kerman and Zagros areas. **F,** endemic to the Iranian Plateau. Distribution maps in **B–F** show cumulative distributions of all considered species pertaining to the respective distribution pattern (see Table 3 for species list and indication of each species’ distribution).

A total of 125 plant species endemic to the Iranian Plateau are recorded from Shirkuh Mts. above 1400 m a.s.l. (Fig. 6A), with 13 of those being local endemics (Fig. 6B). The full species list and their distributions in different mountain ranges are presented in Table 3. Of the 125 species, 95 species are also recorded from Zagros, 70 species from the Kerman massif, 31 species from Alborz, 20 species from the Azerbaijan Plateau, and 14 species from Kopet Dagh‐Khorassan (Fig. 6A). Of these 125 endemic species, 9 species are restricted to the Yazd‐Kerman area (Fig. 6C), 27 species are distributed in the Shirkuh Mts. and in Zagros (Fig. 6D), 30 species are found in the Yazd‐Kerman and Zagros (Fig. 6E), and 11 species are widely distributed in the Iranian Plateau (Fig. 6F). These data show that Shirkuh Mts. are not only a biodiversity hotspot with a rich local endemism, but they are also floristically well connected to the mountain ranges of the Iranian Plateau. This connection is stronger with the geographically close ranges of Zagros and the Kerman massif (Fig. 6A).

**Table 3:** Endemic species of the Iranian Plateau found in Shirkuh Mts. (above 1400 m a.s.l.), their distribution in different areas of endemism, and their presence in the alpine zone (i.e., above 3500 m a.s.l.) of Shirkuh Mts.

Species	Family	Presence in areas of endemism and beyond^a^	Presence in alpine zone
*Allium austroiranicum* R.M.Fritsch	Alliaceae	Za, Sh	X
*Allium cathodicarpum* Wendelbo	Alliaceae	Za, Sh, Ke	X
*Allium jesdianum* Boiss. & Buhse	Alliaceae	Za, Sh, Ke	X
*Ferula hirtella* Boiss.	Apiaceae	Sh, Lo	
*Ferulago contracta* Boiss. & Hausskn.	Apiaceae	Za, Sh	
*Pimpinella dichotoma* (Boiss. et Hausskn.) Wolff in Engler	Apiaceae	Za, Sh	
*Prangos cheilanthifolia* Boiss.	Apiaceae	Al, Za, Sh, Ke	
*Semenovia frigida* (Boiss.) Hausskn.	Apiaceae	Za, Sh	X
*Thecocarpus meifolius* Boiss.	Apiaceae	Za, Sh	
*Trachydium depressum* Boiss.	Apiaceae	Al, Za, Az, Sh, Ke	X
*Alrawia bellii* (Baker) K.Perss. & Wendelbo	Asparagaceae	Za, Sh, Ke	
*Centaurea ispahanica* Boiss.	Asteraceae	Za, Sh, Ke	
*Cirsium spectabile* DC.	Asteraceae	Za, Sh, Ke	X
*Cousinia eriobasis* Bunge	Asteraceae	Za, Sh	
*Cousinia lasiolepis* Boiss.	Asteraceae	Za, Sh	X
*Cousinia longifolia* C.Winkl. & Bornm.	Asteraceae	Za, Sh, Ke	X
*Cousinia onopordioides* Ledeb.	Asteraceae	Al, Sh, Ke, Ko	
*Cousinia sicigera* C.Winkl. & Bornm.	Asteraceae	Sh, Ke	X
*Crepis heterotricha* DC.	Asteraceae	Al, Za, Sh, Ke	X
*Echinops ceratophorus* Boiss.	Asteraceae	Za, Sh, Ke	
*Echinops cervicornis* Bornm.	Asteraceae	Sh	
*Echinops jesdianus* Boiss.	Asteraceae	Sh, Ke	
*Echinops tenuisectus* Rech.f.	Asteraceae	Za, Sh, Ke	
*Helichrysum davisianum* Rech.f.	Asteraceae	Sh	X
*Helichrysum leucocephalum* Boiss.	Asteraceae	Za, Sh, Ke, Bl, Lo	
*Hertia angustifolia* (DC.) O.Kuntze	Asteraceae	Al, Za, Sh, Ke	
*Iranecio paucilobus* (DC.) B.Nord.	Asteraceae	Al, Za, Az, Sh	X
*Jurinea stenocalathia* Rech.f.	Asteraceae	Sh, Lo	
*Launaea acanthodes* (Boiss.) O.Kuntze	Asteraceae	Al, Za, Sh, Ke, Ko, Bl, Lo	
*Scorzonera intricata* Boiss.	Asteraceae	Za, Sh, Ke, Bl	X
*Senecio kotschyanus* Boiss.	Asteraceae	Za, Sh	X
*Tanacetum persicum* (Boiss.) Mozaff.	Asteraceae	Al, Za, Az, Sh, Ko	X
*Taraxacum roseum* Bornm.	Asteraceae	Al, Za, Sh, Ko	
*Tragopogon jezdianus* Boiss. & Buhse	Asteraceae	Al, Za, Sh, Ko	
*Heliotropium aucheri* DC.	Boraginaceae	Za, Sh, Ke	
*Nonea persica* Boiss.	Boraginaceae	Al, Za, Az, Sh, Ke	X
*Onosma stenosiphon* Boiss.	Boraginaceae	Al, Az, Sh, Ke, Ko	X
*Aethionema umbellatum* (Boiss.) Bornm.	Brassicaceae	Za, Sh	X
*Alyssum muelleri* Boiss. & Buhse	Brassicaceae	Az, Za, Sh	X
*Fibigia umbellata* (Boiss.) Boiss.	Brassicaceae	Al, Za, Sh, Ke	X
*Graellsia saxifragifolia* (DC.) Boiss.	Brassicaceae	Za, Sh, Ke, Ko	X
*Hesperis leucoclada* Boiss.	Brassicaceae	Za, Sh	
*Isatis campylocarpa* Boiss.	Brassicaceae	Za, Sh	
*Matthiola ovatifolia* (Boiss.) Boiss.	Brassicaceae	Al, Za, Az, Sh, Ke	
*Pseudocamelina camelinae* N. Busch	Brassicaceae	Za, Sh, Ke	X
*Pseudofortuynia leucoclada* (Boiss.) Khosravi	Brassicaceae	Za, Sh, Ke	X
*Zerdana anchonioides* Boiss.	Brassicaceae	Za, Sh, Ke	X
*Arenaria persica* Boiss.	Caryophyllaceae	Za, Sh	X
*Gypsophila yazdiana* Falat., F.Ghahrem. & Assadi	Caryophyllaceae	Sh	
*Silene daenensis* Melzh.	Caryophyllaceae	Za, Sh	X
*Silene goniocaula* Boiss.	Caryophyllaceae	Za, Az, Sh	X
*Silene gynodioica* Ghaz.	Caryophyllaceae	Al, Za, Az, Sh, Ke, Ko	X
*Silene nurensis* Boiss. & Hausskn.	Caryophyllaceae	Za, Sh, Ke	X
*Yazdana shirkuhensis* A.Pirani & Noroozi	Caryophyllaceae	Sh	X
*Colchicum varians* (Freyn & Bornm.) Dyer	Colchicaceae	Za, Sh	
*Sedum kotschyanum* Boiss.	Crassulaceae	Za, Sh, Ke	X
*Euphorbia connata* Boiss.	Euphorbiaceae	Sh, Ke	
*Euphorbia erythradenia* Boiss.	Euphorbiaceae	Za, Sh, Ke	
*Euphorbia malleata* Boiss.	Euphorbiaceae	Za, Sh	X
*Astragalus albispinus* Sirj. & Bornm.	Fabaceae	Za, Sh, Bl	
*Astragalus anserinaefolius* Boiss.	Fabaceae	Za, Sh, Ke, Bl, Lo	
*Astragalus calliphysa* Bunge	Fabaceae	Za, Sh, Ke	
*Astragalus cephalanthus* DC.	Fabaceae	Za, Sh, Ke	
*Astragalus daenensis* Boiss.	Fabaceae	Za, Sh, Ke	X
*Astragalus darrehbidensis* Podlech & Zarre	Fabaceae	Sh	
*Astragalus glaucacanthus* Fisch.	Fabaceae	Al, Za, Sh, Ke, Lo	
*Astragalus griseus* Boiss.	Fabaceae	Za, Sh, Lo	
*Astragalus horridus* Boiss.	Fabaceae	Za, Sh	X
*Astragalus impexus* Podl.	Fabaceae	Za, Sh	
*Astragalus ischredensis* Bunge	Fabaceae	Za, Sh	
*Astragalus issatissensis* Maassoumi & Mahmoodi	Fabaceae	Sh	X
*Astragalus johannis* Boiss.	Fabaceae	Za, Sh	X
*Astragalus longistylus* Bunge	Fabaceae	Al, Za, Sh, Ke, Bl	
*Astragalus lycioides* Boiss.	Fabaceae	Al, Sh, Ko, Ke	X
*Astragalus mehrizianus* Podlech & Maassoumi	Fabaceae	Sh	
*Astragalus melanocalyx* Boiss. & Buhse	Fabaceae	Za, Sh	
*Astragalus melanodon* Boiss.	Fabaceae	Za, Sh, Ke	X
*Astragalus microphysa* Boiss.	Fabaceae	Za, Sh	X
*Astragalus myriacanthus* Boiss.	Fabaceae	Za, Sh, Ke, Bl	X
*Astragalus pseudoshebarensis* Podlech	Fabaceae	Za, Sh, Ke	
*Astragalus rhodosemius* Boiss. & Hausskn.	Fabaceae	Za, Az, Sh, Ke	
*Astragalus spachianus* Boiss. & Buhse	Fabaceae	Za, Sh, Ke, Ko	X
*Astragalus tenuiscapus* Freyn & Bornm.	Fabaceae	Za, Sh, Ke	X
*Astragalus trachyacanthos* Fischer	Fabaceae	Al, Az, Sh, Ke	
*Astragalus yazdii* (Vassilcz.) Podlech & Maassoumi	Fabaceae	Za, Sh, Ke	X
*Cicer spiroceras* Jaub. & Spach	Fabaceae	Za, Sh, Ke, Bl	
*Onobrychis plantago* Bornm.	Fabaceae	Sh, Ke	X
*Oxytropis shirkuhi* Vassilcz.	Fabaceae	Sh	X
*Oxytropis yazdi* Vassilcz.	Fabaceae	Sh	X
*Ajuga chamaecistus* Ging. ex Benth.	Lamiaceae	Al, Za, Az, Sh, Ke	
*Hymenocrater yazdianus* Rech.f.	Lamiaceae	Za, Sh, Ke	X
*Lagochilus macracanthus* Fisch. & C.A.Mey.	Lamiaceae	Al, Za, Sh, Ko, Lo	
*Nepeta asterotricha* Rech.f.	Lamiaceae	Sh	
*Nepeta bakhtiarica* Rech.f.	Lamiaceae	Za, Sh, Ke	
*Nepeta crassifolia* Boiss. & Buhse	Lamiaceae	Al, Za, Sh, Ko	
*Nepeta gloeocephala* Rech.f.	Lamiaceae	Za, Sh	
*Phlomis aucheri* Boiss.	Lamiaceae	Za, Sh	
*Salvia eremophila* Boiss.	Lamiaceae	Za, Sh, Ke, Lo	
*Satureja bachtiarica* Bunge	Lamiaceae	Za, Sh, Ke	
*Scutellaria multicaulis* Boiss.	Lamiaceae	Za, Az, Sh, Ke	X
*Stachys asterocalyx* Rech.f.	Lamiaceae	Za, Sh	
*Stachys obtusicrena* Boiss.	Lamiaceae	Za, Sh	X
*Thymus carmanicus* Jalas	Lamiaceae	Al, Za, Az, Sh, Ke	X
*Thymus daenensis* Celak.	Lamiaceae	Al, Za, Az, Sh	
*Fritillaria zagrica* Stapf	Liliaceae	Za, Az, Sh	
*Telephium eriglaucum* Williama	Paronychiaceae	Za, Sh	
*Acantholimon festucaceum* (Jaub. & Spach) Boiss.	Plumbaginanceae	Al, Za, Az, Sh, Ke	
*Acantholimon horridum* Bunge	Plumbaginaceae	Sh	
*Acantholimon incomptum* Boiss. & Buhse	Plumbaginaceae	Al, Sh, Ke, Lo	
*Acantholimon kermanense* Assadi & Mirtadz.	Plumbaginaceae	Sh, Ke	X
*Acantholimon modestum* Bornm. ex Rech.f. & Schiman‐Czeika	Plumbaginaceae	Sh, Ke	X
*Acantholimon nigricans* Mobayen	Plumbaginaceae	Za, Sh	X
*Acantholimon scorpius* (Jaub. & Spach) Boiss.	Plumbaginaceae	Al, Za, Az, Sh, Ke	
*Dionysia curviflora* Bunge	Primulaceae	Sh	X
*Dionysia khatamii* Mozaff.	Primulaceae	Sh	X
*Dionysia revoluta* Boiss.	Primulaceae	Za, Sh, Ke	X
*Ranunculus aucheri* Boiss.	Ranunculaceae	Al, Za, Sh, Ke	
*Ranunculus eriorrhizus* Boiss. & Buhse	Ranunculaceae	Sh, Ke	X
*Ranunculus papyrocarpus* Rech.f.	Ranunculaceae	Sh, Ke	
*Cotoneaster persicus* Pojark.	Rosaceae	Za, Sh, Ke	
*Potentilla nuda* Boiss.	Rosaceae	Al, Za, Az, Sh, Ke, Ko	X
*Haplophyllum glaberrimum* Bunge ex Boiss.	Rutaceae	Sh, Lo	
*Salix issatissensis* Maassoumi, Moeeni & Rahiminejad	Salicaceae	Za, Sh, Ke	
*Chaenorhinum grossecostatum* Speta	Scrophulariceae	Sh, Ke	X
*Scrophularia frigida* Boiss.	Scrophulariaceae	Al, Za, Az, Sh, Ke, Ko	X

^a^ Al, Alborz; Az, Azerbaijan Plateau; Bl, Baluchestan; Ke, Kerman; Ko, Kopet Dagh‐Khorassan; Lo, Lowland; Sh, Shirkuh; Za, Zagros.

Of the 13 species endemic to Shirkuh Mts., two species are distributed mainly between 1400 and 2000 m a.s.l. (*Acantholimon horridum* Bunge, Plumbaginaceae; *Echinops cervicornis* Bornm., Asteraceae), three species between 2000 and 2500 m a.s.l. (*Astragalus darrehbidensis* Podlech & Zarre and *Astragalus mehrizianus* Podlech & Maassoumi, both Fabaceae; *Dionysia khatamii* Mozaff., Primulaceae), three species between 2500 and 3000 m a.s.l. (*Dionysia curviflora* Bunge, Primulaceae; *Gypsophila yazdiana* Falat., F. Ghahrem. & Assadi, Caryophyllaceae; *Nepeta asterotricha* Rech.f., Lamiaceae), one species between 3000 and 3500 m (*Helichrysum davisianum* Rech.f., Asteraceae), and four species between 3500 and 4050 m a.s.l. (*Astragalus issatissensis* Maassoumi & Mahmoodi, *Oxytropis shirkuhi* Vassilcz, and *Oxytropis yazdi* Vassilcz., all three Fabaceae; *Y. shirkuhensis* A. Pirani & Noroozi, Caryophyllaceae). *A. issatissensis* was described as a new species (Mahmoodi et al., 2013) from material collected during the same field trip in 2012 when *Y. shirkuhensis* was collected for the first time.

## 4 Conclusion

In this study, *Yazdana*, comprising the sole species *Y. shirkuhensis* from the high alpine zone of the Shirkuh Mts, is introduced as a new genus of Caryophyllaceae. *Yazdana* is closely related to *Cyathophylla* and *Heterochroa*, and thus belongs to a group within Caryophyllinae, whose internal relationships and thus taxonomy have been poorly understood until now (Madhani et al., 2018).

Finding two species new for science (*Y. shirkuhensis* and *Astragalus issatissensis*) in a single trip demonstrates that the plant diversity of this area is still poorly explored. Therefore, detailed studies of flora and vegetation of the Shirkuh Mts., especially in high elevations, are highly recommended. Naturally, the area size decreases sharply from lowlands to high elevations. Whereas plant diversity increases until mid elevation (ca. 2000 m a.s.l.), it decreases gradually until the nival zone (Noroozi et al., 2018). In Shirkuh Mts., however, the number of local endemic species does not decrease with increasing elevation, which could be due to the isolation of higher elevations fostering allopatric speciation. Moreover, of the 125 endemic species of the Iranian Plateau present in Shirkuh Mts., nearly half, that is, 59, species are found at elevations above 3500 m a.s.l. (Table 3). Also, of the 22 species recorded to accompany *Yazdana* (Table 2), 13 species are restricted to Yazd‐Kerman area or Zagros plus Yazd‐Kerman area. As Shirkuh Mts. constitute a “priority conservation gap”, which means, a center of endemism that is currently not within any protected area (Noroozi et al., 2019a), we suggest protecting the area as efficiently as possible to conserve its unique and vulnerable biodiversity.

## Conflict of interest

The authors declare that the research was conducted in the absence of any commercial or financial relationships that could be construed as a potential conflict of interest.

## Supporting information

The following supplementary material is available online for this article at http://onlinelibrary.wiley.com/doi/10.1111/jse.12575/suppinfo:

**Doc. S1.** Voucher information: species name, geographical origin, collector(s), voucher (herbarium), GenBank accession numbers for ITS and *rps16*, respectively. Species names follow the taxonomic treatment suggested in the present study.

**Doc. S2.** All phylogenetic trees obtained from three datasets using Maximum likelihood and Bayesian approaches.

Supporting information.

Supporting information.

Supporting information.

Supporting information.

Supporting information.
