# Supplementary material for: The new locally endemic genus Yazdana (Caryophyllaceae) and patterns of endemism highlight the high conservation priority of the poorly studied Shirkuh Mountains (central Iran)
Source: J Syst Evol. 2020 Mar 24;58(3):339–53. doi: 10.1111/jse.12575 (PMC7318547; doi:10.1111/jse.12575)
Supplement: Supplementary file 1 — Doc. S1. Voucher information: species name, geographical origin, collector(s), voucher (herbarium), GenBank accession numbers for ITS and rps16, respectively. Species names follow the taxonomic treatment suggested in the present study. [file JSE-58-339-s001.docx]

**Supporting Information 1.** Voucher information: species name, geographical origin, *collector(s)*, *voucher* (herbarium), GenBank accession numbers for ITS and *rps16*, respectively. Species names follow the taxonomic treatment suggested in the present study.

***Acanthophyllum aphananthum1*** Rech.f., Afghanistan, Kabul, *Rechinger 31265* (M), KF924626, KF924681; ***Acanthophyllum aphananthum2*** Rech. f., Afghanistan, Prov. Kabul, Kabul, Band-I Kharghak, 34 32 N 69 06 E, 2050 m, 24.6.1965, Rechinger 31265 (MSB) –, MF401175; ***Acanthophyllum allochrusoides1*** (Gilli) Pirani, Afghanistan, Bamian, *Wendelbo & Ekberg W9801* (GB), KF924627, KF924682; ***Acanthophyllum allochrusoides2*** (Gilli) Pirani, Afghanistan, Prov. Baghlan, Andarab-Tal, Fuβ des Koh-e Shindadara bei Shashan (NE von Deh Salah), 1900-2000 m, 35 47 N 69 21 E, 28.5.1972, O. Anders 9367 (MSB), –, MF401172; ***Acanthophyllum borsczowii*** Litv., Iran, Khorassan, *Zarre & al. 41034* (TUH), KF924675, KF924727; ***Acanthophyllum bungei1*** (Boiss.) Trautv., Iran, E Azarbaijan, *Rechinger 43834* (M), KF924634, KF924688; ***Acanthophyllum bungei2*** Boiss., Turkey, A9 Kars, Kaĝizman-Tuzluça, 13 km W Tuzluça, 1060 m, 30.7.1984, Nydegger 19519 (MSB), MF401121, MF401169; ***Acanthophyllum*** ***cerastioides1*** (D.Don) Madhani & Zarre, Pakistan, Hazar, *Ewald & Zetterlund 6227* (GB), KF924628, KF924683; ***Acanthophyllum*** ***cerastioides2*** (D.Don) Madhani & Zarre, NW Pakistan, Swat, in valle Jabba E Kolalai, substr. Granit, 1600-2200 m, 4.6.1965, *Rechinger 30724* (M), MF401122, MF401168; ***Acanthophyllum grandiflorum*** Stocks, Afghanistan, Bamian, *Podlech 1340* (MSB), KF924666, KF924718; ***Acanthophyllum herniarioides*** (Boiss.) Madhani & Zarre, Tajikistan, N von Dushanbe Anzob-Pass im Gissar-Massiv, ca. 3400 m, 14.7.1975, *Mueller-Doblies 75083* (B), MF401123, –; ***Acanthophyllum honigbergeri*** (Fenzl) Barkoudah, E Afghanistan, Gardes, in altoplanitie lapidosa vallis Logar 50 km N Gardez, 33 37 N, 69 09 E, 2000 m, *Rechinger 35371* (B), MF401125, MF401176; ***Acanthophyllum kabulicum*** Schiman-Czeika, Afghanistan, Ghazni, *Frey s.n.* (GB), KF924663, KF924715; ***Acanthophyllum kandaharicum*** Gilli, Iran, Khorassan, *Joharchi & Zangouei 36245* (FUMH), KF924662, KF924714; ***Acanthophyllum laxiflorum*** Boiss., Afghanistan, Lugar, *Ekberg W9184* (GB), KF924659, KF924711; ***Acanthophyllum mucronatum1*** C.A.Mey., Iran, W Azarbaijan, *Assadi & Olfat 68668* (TARI), KF924652, KF924705; ***Acanthophyllum mucronatum2*** C.A.Mey., Armenia, Vayotsdzor prov., Yeghegnadzor, vicinity of Agarakadzor villag, Azar gorge, 1320 m, 26.6.2002, Optima Iter XI/2050 (M), –, MF401170; ***Acanthophyllum myrianthum*** (Rech.f.) Madhani & Pirani, C-Afghanistan, Deh Kundi: in saxosis 10 kn w Shahrestan, 33 40’N, 66 35’E, versus Deh Kundi, 34 10’ N 66 07’ E, 2200 m, 1967, *Rechinger 36812* (B), MF401124, –; ***Acanthophyllum oppositiflorum*** Aytaç, Turkey, Sivas, *Aytaç 7476* (GAZI), KF924651, KF924704; ***Acanthophyllum scapiflorum*** (Akhtar) Schiman-Czeika, Afghanistan, Kabul, *Podlech 31232* (MSB), KF924646, KF924699; ***Acanthophyllum sordidum*** Bunge ex Boiss., Iran, Isfahan, *Pirani & Moazzeni 2147* (TMRC), KF924644, KF924697; ***Acanthophyllum spinosum*** C.A.Mey., Iran, Isfahan, *Pirani & Moazzeni 2150* (TMRC), KF924642, KF924696; ***Acanthophyllum stocksianum*** Boiss., Afghanistan, Kandahar, *Toncev s.n.* (MSB), KF924639, KF924693; ***Acanthophyllum versicolor*** Fisch. & C.A.Mey., Turkey, Kars, *Nydegger 43597b* (MSB), KF924633, KF924687; ***Arenaria serpylloides*** Gay, KP148897, KP148997; ***Balkana spergulifolia*** (Griseb.) Madhani & Zarre, Serbia, Altserbien, Zlatibor, südlich von Kremna, Umgebung von Gaj, Cigota Höhen, 1020-1475 m,15.9.2004, *Kalheber 04-1558* (M), MF401126, MF401185; ***Bolanthus cherlerioides*** (Bornm.) Bark., Turkey, B3 Isparta, Akşehir-Şarkikaraağaç, 5.1 km NE Örkenez an der Paßauffahrt, Schutt, 1480 m, 12.8.1975, *K.P. & E. Buttler 19986* (M), MF401128, MF401183; ***Bolanthus confertifolius*** (Hub.-Mor.) Madhani & Heubl, Turkey, Antalya, between Fethiye and Kalkan, *Pinus brutia* forest, c. 200 m, 5.2002, *Özkan Eren 4362* (B), KX834007, –; ***Bolanthus huber-morathii*** Simon, Turkey, A2 Bursa, Soĝukpinar-Keles, 4 km nach Soĝukpinar, 860 m, 5.7.1980, *Nydegger 15138* (MSB), KX834006, MF401184; ***Bolanthus minuartioides*** (Jaub. & Spach) Hub.-Mor. Turkey, Denizli, Muğla, 6.6.1955 , *Walter 201* (B), KX834005, –; ***Bolanthus ortegioides*** (Fisch. & C.A.Mey.) Madhani & Rabeler, Turkey, B5 Kayseri, above Talas, Ağida mt., 1700 m, 8.8.1997, *Zarre 42* (MSB), KX834008, MF401182; ***Cerastium fontanum*** Baumg., AY936241, FJ404899; ***Cyathophylla chlorifolia*** (Poir.) Bocquet & A. Strid, Turkey, C3 Antalya, SW Anatolien, offener Steinschutt über Salikent, 2050 m, Exp. N, 2.9.1995, *Ulrich s.n.* (M), –, MF401186; ***Cyathophylla viscosa*** (C.A.Mey.) Madhani & Rabeler, Armenia, Vayotsdzor prov., Vajk distr, road Vajk-Kochbek, ca. 8 km ENE Vajk, gorge of Darab river, 1380 m, sandy area, 1380 m, 26.6.2002, *Optima Iter XI/1846* (M), MF401117, MF401165; ***Dianthus andrzejowskianus*** Kulcz., JN589032, –; ***Dianthus armeria*** L., JN589087, FJ404903; ***Dianthus candicus*** (Ball & Heywood) Madhani & Heubl, Greece, Ep. Sfakia, in declivibus australibus montis Akones ad orientem pagi Imvros, 1100-1150 m, 8.10.1966, *Greuter 7679* (M), –, MF401178; ***Dianthus carthusianorum*** L., EF407941, EF674194; ***Dianthus chinensis*** L., JN589157, –; ***Dianthus cyri*** Fisch. & C.A.Mey., GU440808, –; ***Dianthus deltoides*** L., JN589027, –; ***Dianthus recticaulis*** Ledeb., Armenia, Vayotsdzor prov., Yeghegnadzor distr., ca. 12 km N Yeghegnadzor, around village Eghegis, 1540 m, 45 22 E, 39 52 N, humid meadows along river, forest with Quercus macranthera, dry slopes and rocks, 27.6.2002, *Fayvush et al., OPTIMA Iter XI/2199* (M), –, MF401177; ***Dianthus tunicoides*** (Ser.) Madhani & Heubl,, Greece, Chios, ca. 2 km nordöstlich Volissos am Rand der Straβe nach Potamia, Grauwacke, 200 m, 16.6.1966, *Lüdtke 581* (M), MF401129, MF401179; ***Diaphanoptera afghanica*** Podlech, Afghanistan, Baghlan, *Podlech 21075* (MSB), KF924632, –; ***Diaphanoptera ekbergii1*** Hedge & Wendelbo, Afghanistan, Takhar, *Podlech 11848* (MSB) & *11760* (MSB), KF924631, KF924686; ***Diaphanoptera ekbergii2*** Hedge & Wendelbo, Afghanistan, Prov. Takhar, Khost-o-Fereng, oberes Khaush-Tal; Granitfelsen, 3800 m, 11.7.1965, Podlech 11760 (MSB), –, MF401173; ***Diaphanoptera lindbergii*** Hedge & Wendelbo, Afghanistan, Fariab, *Hedge & al. W8336* (GB), KF924630, KF924685; ***Diaphanoptera stenocalycina1*** Rech.f. & Schiman-Czeika, Iran, Golestan, *Attar & Mehdigholi 24422* (TUH), KF924629, KF924684; ***Diaphanoptera stenocalycina2*** Rech.f. & Schiman-Czeika, Iran, Prov. Golestan, Golestan National Park, Almeh, 1600 m, 19.5.1975, Firuznia 1174 (M), -, MF401174; ***Eremogone aculeata*** (S.Watson) Ikonn., JN589018, FJ404882; ***Eremogone picta*** (Sm.) Dillenb. & Kadereit, KP148933, KP149035; ***Graecobolanthus fruticulosus*** (Bory & Chaub.) Madhani & Zarre, Greece, Insula Euboea septentrionalis, in saxosis serpentinicis et manesiticis ad litus a pago Mantudi orientem versus, 30.6.1958, *Rechinger 19439* (M), –, MF401180; ***Graecobolanthus graecus*** (Schreb.) Madhani & Rabeler, Greece, Epirus, Tal des Venetikos nördlich Eleftherokhori, an der Straβe Kalambaka-Grevena, 500 m, 11.10.1975, *Merxmüller & Podlech 31173* (MSB), KX834004, MF401181; ***Gypsophila acantholimoides*** Bornm., Iran, Kuh-i Karkas (Kuh-i Kargiz), in declivibus supra Tar, 2300-2500 m, 27.5.1974, *Rechinger 46581* (MSB), MF401083, MF401141; ***Gypsophila acutifolia*** Fisch., Russia, Podkumok-Tal bei Kislovodsk, 21.7.1967, *Quasdorf 67* (B), MF401100, MF401156; ***Gypsophila antari*** Post, Iraq, Distr. Basra, Desertum meridionale (Southern Desert) Jabal Sanam, ca. 30 10 N, 47 30 E, 18.3.1967, *Rechinger 8568* (M), MF401089, MF401134; ***Gypsophila arabica*** Barkoudah, Israel, Negev Highlands: Makhtesh Hazera (Makhtesh Katan), sandy alluvium, pebbly wadis and limestone outcrops, Altim. 10 m, 10.3.1989, *Danin & al. 35.036* (B), MF401082, –; ***Gypsophila aretioides*** Boiss., Iran, Gachsar, Hezar Band mountain, alt. 3200 m, 30.7.2015, 36°03'N 51°17'E, *Madhani 47116* (TUH), MF401090, –; ***Gypsophila arrostii*** Guss., JN589043, –; ***Gypsophila arrostii*** Guss. var. ***nebulosa*** (Boiss. & Heldr.) Greuter & Burdet, Turkey, C3 Afyon, Isparta-Denizli, 21 km SE Dinar, 990 m, 5.8.1978, *Nydegger 13410* (MSB), –, MF401155; ***Gypsophila aucheri1*** Boiss., JN589077, –; ***Gypsophila aucheri2*** Boiss, Turkey, B8 Erzincan, Erzerum und Tercan, 18 km östlich Tercan), 1860 m, Steilbord, 2.8.1983, *Nydegger 18633* (MSB), MF401098, MF401147; ***Gypsophila bermejoi*** G. Lopez, Spain, prov. Segovia, Vallelado, UTM 30t UL 78, alt. 750 m, 26.8.1983, *Ladero & Casaseca 12107* (B), MF401106, –; ***Gypsophila bicolor*** Grossh., JN589151, –; ***Gypsophila bicolor*** (Freyn & Sint.) Grossh., Iran, Prov. Ghazvin, Abgarm to Avaj, 12 km to Avaj, 1 km to tunnel, beside the road, 1420-1500 m, 10.5.2004, *Zarre, Mashayekhi, Taeb, Pirani & Moazzeni 35136* (MSB), –, MF401149; ***Gypsophila bucharica1*** B. Fedtsch., JN589057, –;***Gypsophila bucharica2*** B. Fedtsch., Tadzhikistania, jugum Chodzha-Kazian, declivibus australis montis Koipioztau, 1000 m, 8.5.1976, *Kinzikaeva & Koczkareva 6663* (M), MF401102, MF401162; ***Gypsophila capillaris1*** (Forssk.) C. Chr., KJ021878, –; ***Gypsophila capillaris2*** (Forssk.) C. Chr., Egypt, Sinai Peninsula, Jebel Maghara, 8 km N of Bir el Hamma, 270 m, siliceous rocks and flats, 33 30 E, 30 40 N, 3.5.1991, *Podlech* *50067* (MSB), MF401092, MF401135; ***Gypsophila capitata*** Bieb. Russia: Caucasus, Dagestan, distr. Chunzach, inter pag, Golotl et Kachib, vallis fl. Avarskyi Koissu, 17.7.1961, *Tzvelev, Czerepanov, Bobrov & Dogadova 7559* (B), MF401103, MF401161; ***Gypsophila capituliflora1*** Rupr., JN589143, –; ***Gypsophila capituliflora2*** Rupr., Tadzhikistan: Pamir orientalis, Czeczekty, prope Stationem Biologicam, fundus siccus valleculae, 3850 m, 21.8.1959, *Ikonnikov 4365* (M), MF401111, MF401157; ***Gypsophila cephalotes1*** (Schrenk ex Fisch. & C.A. Mey.) Raikova, JN589105, –; ***Gypsophila cephalotes2*** (Schrenk ex Fisch. & C.A. Mey.) Williams, Afghanistan, Prov. Badakhshan, Wakhan, unteres Waghjir Tal bis Zemestan-e Tikili, 37 06 E, 74 05 N, 3950-400 m, 21.7.1971, *Anders 7613* (MSB), MF401105, MF401158; ***Gypsophila curvifolia1*** Fenzl, JN589159, –; ***Gypsophila curvifolia2*** Fenzl, Turkey, C 4 Antalya, Orta Toroslar, zwischen Anamur und Kazanci, Friedhof bei Akpinar, Hügel aus (Kreide)- Kalk, offene Ruderalflur, 1630 m, 36 20’ N, 32 50’ E, 22.7.1992, *P. Hein 52-2* (B), MF401099, MF401159; ***Gypsophila elegans1*** M.Bieb., JN589130, –; ***Gypsophila elegans2*** M.Bieb., Iran, Prov. Azarbaijan Sharqi, 19 km SE of Asheeqli (Asheglou) in the Aras valley at road to Kaleibar, Arasbaran Protected Area, 1850 m, 46 48 55 E, 38 53 54 N, 1850 m, 20.6.2001, *Podlech & Zarre 55293* (MSB), –, MF401143; ***Gypsophila elegans3*** M. Bieb., Germany, Bayern, Oberpfalz, Weiden, Mülldeponie/Bauschttdeponies, offene Erde, 410-420 m, MTB 6338/2, 28.8.1991, *Weigend 1895* (M), MF401081, MF401144; ***Gypsophila fastigiata1*** L., JN589144, –; ***Gypsophila fastigiata2*** L., Germany, Rheinland-Pfalz, Rheinhessen, Mainz, Autobahnböschung am NSG Großer Sand, 17.7.1988, *Kalheber 88-2892* (M), MF401097, MF401152; ***Gypsophila globulosa*** Stev. Russia, Caucasica, Pjatigorsk, Felshügel über den Mineralquellen, ca. 4-500 m 11.7.64, *Köhler* *(61)* *Bm 4306210* (B), MF401108, –; ***Gypsophila glomerata*** Pall. ex M. Bieb., Bulgaria, Bezirk Kolarovgrad, Kalkfelsen bei Madara, 17.8.1968, *Merxmüller & Zollitsch 24599* (M), MF401109, –; ***Gypsophila gypsophiloides*** (Fenzl) Blakelock, Iran, Prov. Luristan, Dow Rud, in declivibus aridis ad intoritum faucium fluvii Dez, substr. Calc., 1500-1600 m, 17.6.1974, *Rechinger 48149* (M), MF401086, MF401138; ***Gypsophila heteropoda1*** Freyn, JN589110, –; ***Gypsophila heteropoda2*** Freyn, Georgia, Caucasus, peripheria urbis Tbilisi, haud procul a lacu Lisi, Alt. 600 m s. m., 17.5.1985, *Va*šá*k*  *s.n.* (B), MF401085, –; ***Gypsophila laricina*** Schreb., Turkey, C5 Adana, Aladaglari, Kayacik Deresi, entrance to Narpiz Bogazi gorge, northern slope, alt. 2450 m, 7.8.1999, *Doring, Parolly & Tolimir 1231* (B), MF401112, MF401145; ***Gypsophila leioclada*** Rech.f., Iran, Prov. Azarbaijan Sharqi, near At Darrehsi, ca. 70 km SE Bostanabad toward Mianeh, 1434 m, 47 23 23 E, 37 30 54 N, 18.6.2001, *Podlech & Zarre 55219* (MSB), MF401104, MF401148; ***Gypsophila linearifolia*** (Fisch. & C.A.Mey.) Boiss., Iran, Prov. Ilam, 36 km from Dehloran towards Mehran, gypsum hills, Pteropyrum community, 320 m, 4.5.1992, *Akhani 8509* (MSB), MF401091, MF401136; ***Gypsophila nabelekii*** Schischk., Iran, Azerbaijan occidentalis, in monte Chalil Kuh prope Razhan, 2600-3200 m, 1974, *Rechinger 48847* (B), MF401088, MF401142; ***Gypsophila oblanceolata*** Bark., Turkey, Provinz Nigde, bei Eregli nordlich von Ulukila, 1150 m u. m., Steppe, Bodenoberfläche mit Trockenrissen und Salzabscheidungen, 27.9.1984, *Hagemann, Binder & Schwarz 2144* (B), MF401115, MF401160; ***Gypsophila sp.*** (Fisch. et C.A.Mey.) Boiss., Turkey, Akdagh bei Amasya, *Manissadjian 1165* (B), MF401087, –; ***Gypsophila* *pacifica*** Komarov, JX274528, –; ***Gypsophila paniculata*** L., JN589150, FJ404908; ***Gypsophila patrinii1*** Ser., JN589076, –; ***Gypsophila patrinii2*** Ser. Russia, Altay Republic, ongudayskiy Rayon, N side of Chuya valley near Belyy Bom 31 km SSE of Inya- Dry shrubland with Artemisia, S-exposed, limestone, 980 m,50 21’N, 087 03’E, 15.7.2002, *Raab-Straube 020105* (B), MF401110, MF401150; ***Gypsophila perfoliata*** L., Turkey, B4 Ankara, Yassihuyuk (ancient site of Gordion), ancient of Gordion proper. Lat. 39 36’N. long. 032 02’E, alt. c. 1100 m, 30.9.1999, *Doring, Parolly & Tolimir 7438* (B), MF401114, MF401139; ***Gypsophila petraea*** (Baumg.) Rchb., Romania, Reg. Ploiesti Bucegi, vale Jepilor zwischen Busteni cab. Caraman, Felsen, 1600-2000 m, 30.7.1965, *Buttler & Dietrich 8953* (B), MF401095 (ITS1), MF401151; ***Gypsophila pilosa1*** Huds., Spain, Murcia, Provincia de Albacete, Abzweigung von der Straße Hellin-Cieza in Richtung Agramón kurz vor Minateda, Felsen aus miozänen Kalken und Gipsflächen, ca. 520 m, 19.5.1983, *Bayer & Grau 34* (M), MF401093, MF401140; ***Gypsophila pilosa2*** HUDS., Philos., Trans. Iran, Prov. Isfahan, At the entrance of Ghamsar to Ghohroud, ruderal vegetation of main Boullevard 5 km after Ghamsar, Alt. 1667 m, 33° 45' 33.09"N, 51° 28' 31.50"E, 27.05.2015, *Zarre & Madhani 34287* (TUH) MF401094, –; ***Gypsophila pilulifera*** Boiss. & Heldr, JN589132, –; ***Gypsophila pinifolia1*** Boiss. & Hausskn. ex Boiss., JN589050, –; ***Gypsophila pinifolia2*** Boiss. & Hausskn. ex Boiss., Turkey, B6 Malataya, (Straße O, Malataya-36,5-Gürün, 140) c. 4 km E Sarĭhacĭ köyü an der Straße nach Malataya Felshang, 1480 m, 29.8.1971, *Buttler 5774* (M), MF401116, MF401163; ***Gypsophila repens1*** L., KF737521, –; ***Gypsophila repens2*** L., Austria Tirol, Stubaier Alpen, E-Hänge der Serlesschare SW Maria Waldrast bei Matrel a. Brenner, 2100 m, 1.8.1983, *Podlech 38401* (MSB), MF401101, MF401153; ***Gypsophila scorzonerifolia*** Ser., JN589100, –; ***Gypsophila silenoides*** Rupr., JN589049, –; ***Gypsophila stevenii*** Fisch. ex Schrank, JN589022, –; ***Gypsophila tomentosa*** L., Spain, Alicante, El Salobrar, 12.6.1986, *Molero 30SWJ90 (33)* (M), MF401113, MF401146; ***Gypsophila venusta*** Fenzl, Turkey, Anatolia, B6, Sivas, Zwischen Zara und Sivas , 15 km E Sivas, alt. 1250 m, Steppe auf Marmor, 22.7.1981, *Nydegger n 16995 k, n 12109* (B), MF401096, MF401154; ***Gypsophila virgata*** Boiss. Iran, Prov. Azerbaijan orient., in saxosis faucium 38 km NNW Marand versus Jolfa, 1100 m. *Rechinger 43614* (B), MF401107, –; ***Gypsophila viscosa*** Murray. Turkey, B5 Nevsehir, Goreme Tarihi Milli Parkim Goreme Valley, slopes SE of the Open Air Museum, ca. 1050-1100 m, tuff, secondary steppe and ruderal vegetation, 25.5.2006, *Bircan & Parolly 110* (B), MF401084, MF401137; ***Heterochroa desertorum1*** (Bunge) Fenzl, JN589021, –; ***Heterochroa desertorum2*** (Bunge) Fenzl, Russia, Tuviskaja ACCP, Tuva, distr., Ovjur, prope pagum Ak-Czyra cleistogeneto-nanphyteta stepposa, 3.8.1973,  *Timokhina & Daniljuk 6371* (M), MF401118, MF401171; ***Heterochroa violacea*** Fenzl, JN589068, –; ***Moehringia lateriflora*** (L.) Fenzl, JX274536, FJ404924; ***Petroana* *montana*** (Balf.f.) Madhani & Zarre, Aden peninsula, Upper Crater, plateau SE of the Tower of Silence, c. 120-170 m, 12 47’N, 45 02’E, on fine-gravelly Pozzolane, 22.3.1997, *Kilian, Hein & Smalla NK 4487* (B), MF401119, MF401167; ***Petroana montserratii1*** (Fern.Casas) Madhani & Zarre,, JN589155, –; ***Petroana montserratii2*** (Fern.Casas) Madhani & Zarre, Spain, Albacete: pr. Ferez, 800 m, in rupi bus calcareis verticalibus, 19.8.1972, *Casas s.n.* (B), MF401120, MF401166; ***Petrorhagia prolifera*** (L.) P.W.Ball & Heywood, GU440883, –; ***Petrorhagia saxifraga*** (L.) Link, JQ307895, FJ404930; ***Petrorhagia thessala*** (Boiss.) P.W. Ball & Heywood, GU440885, –; ***Petrorhagia dubia*** (Raf.) G. López & Romo, AY857974, –; ***Psammophiliella muralis1*** (L.) Ikonn., JN589037, –; ***Psammophiliella muralis2*** (L.) Ikonn., Germany, Bayern, MTB 6831/1: Acker nahe Mohrhof bei Poppenried/Höchstädt, 25.8.1987, *E. Dörr s.n.* (M), MF401127, MF401186; ***Psammosilene tunicoides*** W.C.Wu & C.Y.Wu, JN589122, –; ***Saponaria griffithiana*** Boiss., Afghanistan, Prov. Badakhshan, Shewa valley, 37 16, 70 38, 2640 m, 29.5.2008, *Schloeder & Jacobs 1757* (M), MF401080, MF401133; ***Saponaria ocymoides1*** L., AY936271, FJ404936; ***Saponaria ocymoides2*** L., Spain, Prov. Teruel, Monte Sierra de Javambre, 1650-1700 m, 9.7.2002, *Šída & Vagnerová 3658* (M), MF401077, MF401130; ***Saponaria officinalis1*** L., AY594313, FJ404937; ***Saponaria officinalis2*** L., Japan, Kamite, Toyoshina-machi, Minami-azumi-gun, Nagano Pref., 550 m, 9.9.2002, dry bed river, *Sugawara 2080906* (M), MF401078, MF401131; ***Saponaria prostrata*** Willd., Turkey, B6 Kahramanmaraş, 30 km to Göksun from Sariy, Bin Boğa Daği, above Yalak village, 2100-2400 m, 11.8.1997, *Zarre 122* (MSB), MF401079, MF401132; ***Saponaria pumila*** Hayek, AY594311, –; ***Saponaria sicula*** Raf., –, Z83153; ***Silene alexandri*** Hillebr., EF060222, EF061382; ***Silene gallica*** L., U30959 (ITS1), U30985 (ITS), JX560214; ***Silene italica*** (L.) Pers., AY936258, KF305909; ***Silene repens*** Patrin, JX274527, DQ908842; ***Silene viscosa*** (L.) Pers., FN821148, FN821316; ***Silene vulgaris*** (Moench) Garcke, AY857967, EF674192; ***Stellaria media*** (L.) Vill., KF737498, FJ404953; ***Vaccaria hispanica1*** (Mill.) Rauschert, X86896.1; ***Vaccaria hispanica2*** (Mill.) Rauschert JF421553.1(ITS2); ***Vaccaria hispanica3*** (Mill.) Rauschert X83847.1(ITS2); ***Velezia rigida1*** L., AY936269(ITS), –; ***Velezia rigida*2** L., GU440888(ITS), –. ***Yazdana shirkuhensis*** Pirani & Noroozi, Iran, Yazd, Shirkuh Mts, Noroozi 2827(WU), MK637517, MK651077.; ***Yazdana shirkuhensis*** Pirani & Noroozi, Iran, Yazd, Shirkuh Mts, Noroozi 4003(WU)MN381230–MN381234, MN417289-MN417292
